# Examining the effect of moderating variables on autonomous public van acceptance model (APVAM)

**DOI:** 10.1371/journal.pone.0290030

**Published:** 2023-08-11

**Authors:** Hossein Naderi, Habibollah Nassiri

**Affiliations:** Civil Engineering Department, Sharif University of Technology, Tehran, Tehran State, Iran; University of Zilina, SLOVAKIA

## Abstract

Autonomous vehicles (AVs) will soon become the primary means of transportation for millions of people. However, the introduction of these vehicles may lead to increased traffic and changes in people’s travel patterns. To address this issue, one solution is to promote the use of AVs in a shared and public manner. However, the success of this approach depends on public acceptance of public AVs. If the promoters of public AVs are unaware of how people perceive this technology, it is possible that these vehicles will face failure in the market. Therefore, this paper aims to identify the factors that influence the willingness to use public AVs. To achieve this, an autonomous public van acceptance model (APVAM) was developed by adapting the unified theory of acceptance and use of technology (UTAUT2) through structural equations modeling. In this study, 824 citizens of Tehran participated in a field study and completed an online questionnaire. The research results indicated that the variable of effort expectancy indirectly affects the use of autonomous public vans (APVs), while the variables of performance expectancy, facilitating condition, hedonic motivation, and perceived PUnTrust directly affect their use. The research also found that various factors such as gender, level of education, individualism/collectivism, travel purpose, the dominant travel mode, marital status, occupation, age, household wealth, number of vehicles owned, and the price of the current vehicle of the household have a moderating effect on the APVAM. Furthermore, it was revealed that individuals who primarily use personal or public transport for their daily trips are more influenced by the perceived enjoyment of APVs in increasing their willingness to use these vehicles, compared to those whose predominant mode of travel is walking or cycling.

## Introduction

The technology of autonomous vehicles (AVs) has been the greatest technological progress in the global transportation industry, offering a promise of a safer and more convenient future. This technology is no longer a fantasy and will become a common means of transportation for millions of people [1–3]. Conducting studies on AVs is crucial to anticipate and address major transformations, maximizing benefits and minimizing adverse consequences such as increased traffic congestion, air pollution, and privacy concerns in shared AVs. Despite uncertainties surrounding AVs in terms of technology, law, and social issues, planning for these challenges before their widespread adoption is more convenient and cost-effective [4].

The success of AVs relies on user acceptance [5], which is a complex process influenced by various factors. User acceptance refers to the users’ willingness to adopt and use the technology and its applications [6]. User acceptance of AVs requires studies to identify the factors affecting the acceptance of these vehicles before their availability to the public. Despite studies conducted in this area, there is still limited knowledge about the acceptance of AVs in international communities particularly in developing countries like Iran.

Kyriakidis et al. indicated that identifying the attitude of people, drivers of non-AVs, and other users of the roads about the technology of AVs is significant. They called for further research and studies in this area [7]. Over recent years, several researchers (e.g. [3, 7–10]) have conducted field studies to understand the public perception about the advantages and limitations of AVs. Although these studies have provided descriptive statistics associated with public awareness, as well as the concerns and advantages expected by autonomous vehicle (AV) technologies, they have less explained the interrelationships between the variables affecting the tendency of users to ride AVs as well as exploring the effect of moderating variables. Furthermore, there has been limited research on the acceptance of AVs in specific contexts such as public transport, including autonomous public vans (APVs).

Therefore, this research aims to accomplish the following objectives by studying the behavior and opinions of citizens in Tehran (the capital of Iran) regarding fully automated APVs (Level 5 according to the society of automotive engineers (SAE) standard [11]):

1. Develop an acceptance model (tendency to use) for APVs as a specific type of autonomous vehicles.
2. Study and explore the effects of moderating variables on the autonomous public van acceptance model (APVAM) constructs.

To achieve these objectives, the APVAM has been developed in the main model and hypotheses section based on previous studies. Then through structural equations modeling, the final and validated model of autonomous van acceptance has been introduced, followed by the examination of the impact of the moderating variables.

## Conceptual research model and hypotheses development

### Literature review

In general, studies on the acceptance of AVs can be categorized into three main groups. First, the technology acceptance model (TAM) introduced by Davis in 1985 to identify people’s intentional behavior to use emerging technologies. TAM includes two variables, perceived usefulness and perceived ease of use, which influence technology acceptance [12]. This model developed to identify acceptance of computer-based information systems and was later used in the field of AV acceptance studies (e.g. [13, 14]). Payre et al. conducted a study to investigate the impact of attitude and a priori acceptability on the willingness to use fully automated vehicles. The study involved 421 French drivers who completed an online questionnaire. The results indicated that French drivers are more inclined to use fully automated cars on highways, in traffic jams, and for parking [13]. Another study by Choi and Ji utilized the TAM model to identify the factors influencing people’s trust in AVs. The findings revealed that perceived usefulness and trust have the most significant effect on increasing the desire to use these cars [14]. Some researchers have developed new models based on TAM. For example, Nees created the self-driving car acceptance scale (SCAS) based on TAM. Nees presented a 12-item measurement scale to evaluate the acceptance of self-driving cars and concluded that showing explanatory images of self-driving cars to participants increases their intention to use these cars [15].

Another widely used acceptance model in studies on the acceptance of AVs is the unified theory of acceptance and use of technology (UTAUT2) [16]. Several researchers have developed AV acceptance models based on the UTAUT2, considering different types of self-driving cars and cultural contexts (e.g. [17, 18]). For instance, in a study on the acceptance of driverless shuttles, Nordhoff et al. found that perceived usefulness, ease of use, pleasure, and trust in driverless vehicles influenced the willingness to use these vehicles. However, they did not find a significant correlation between socio-economic variables and the acceptance of these vehicles [17].

In the third category of studies on the acceptance of AVs, researchers have deviated from conventional acceptance models. Instead, they have focused on identifying people’s opinions, views, and concerns regarding AVs (e.g. [19–22]). For instance, Bazilinskyy et al. concluded that individuals can be categorized into two groups: optimistic and pessimistic towards AVs, with a small number having a neutral stance [19]. Liljamo et al. examined attitudes and concerns about AVs among 2,036 Finnish citizens. The findings revealed that men, individuals with higher education, people residing in densely populated areas, and those without cars in their households had a more positive view towards AVs [21]. In 2016, Zmud et al. explored acceptance and travel behavior influenced by AVs. The results indicated that 50% of the participants expressed a willingness to use AVs for daily trips, and 59% preferred personal AVs over shared ones. Furthermore, 23% of the participants stated that they would no longer own their current car if AVs were available in the market [22].

Additionally, some studies investigated the acceptance of different models and applications of AVs. For instance, Winter et al. examined patients’ perceptions of Driverless ambulance. In this study, the researchers compared patients’ willingness to ride in a fully automated ambulance versus an ambulance with a human driver. The findings indicated that patients were less inclined to ride in driverless ambulances compared to those with human drivers, with women being more hesitant than men [23].

By reviewing the literature, it becomes clear that there are three research gaps in AV acceptance studies. Firstly, there have been few studies examining the acceptance of AVs among citizens in developing countries. Secondly, most previous studies have not investigated the effect of moderator variables on the acceptance model of AVs, as this issue has been explicitly mentioned in Keszey et al. study [24]. lastly, no studies specifically examined the acceptance of autonomous public vans (a specific type of shared AV). So, this study has three innovations compared to previous studies to fulfill the previous research gaps, including the following:

- Introducing the APVAM as an extension of UTAUT2 by adding new variables, such as perceived untrust and perceived risk in developing countries.
- Studying the effect of 12 new moderating variables on the APVAM, such as individualism/collectivism, demographic variables, etc.
- Studying the acceptance of a specific type of autonomous vehicle: the public van.

### Main model and hypotheses

**Fig 1:**
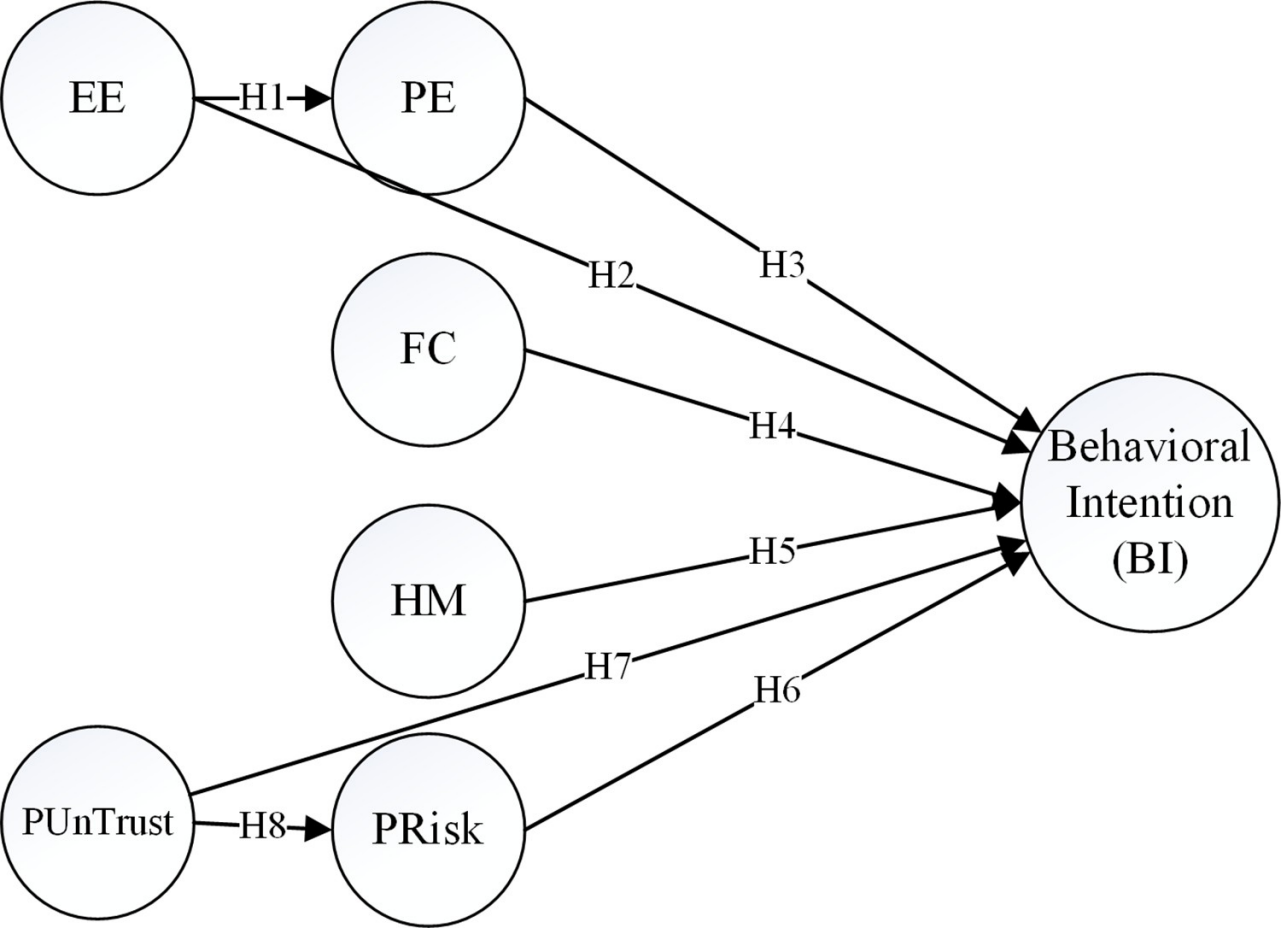
Proposed autonomous public van acceptance model (APVAM).

In developing the intended APVAM model for this research, UTAUT2 served as the initial foundation [25, 26]. The conceptual model of accepting APVs in this study has been developed in line with the conditions of the Iranian population and the type of AVs explored in this study, i.e. public van vehicles. The model proposed in this study, named the "autonomous public van acceptance model (APVAM)", is depicted in Fig 1.

In this model, performance expectancy (PE) refers to the extent of effectiveness of an autonomous van in fulfilling the benefits of people in line with their needs. The performance expectancy indicators in the UTAUT2 model are very similar to the indices of the perceived usefulness construct in the TAM presented by Davis [12]. Indeed, it can be stated that the performance expectancy construct has been adapted from the perceived usefulness construct.

Effort expectancy (EE) represents the ease of use for users when interacting with APVs. The indices of this latent variable have great similarity to the indices of perceived ease of use construct in the TAM [12].

Facilitating conditions (FC) in this model reflect participants’ perception of the level of resources and support available for using APVs. Another construct that has been used in this model is hedonic motivation (HM), which means the extent of fun and pleasure the users of an autonomous van will gain [26].

As mentioned earlier, some of the constructs introduced in the UTAUT2 model such as social influence (SI), habits, and price value [26] have not been presented in the APVAM model.

The SI construct measures the influence of others’ opinions on the respondents’ use of APVs. However, since APVs are a new technology in themselves and the perception of many participants about the APVs is merely limited to the introduction of these vans at the beginning of this survey, response about the opinions of friends and acquaintances regarding AVs is complex and unreliable. During the pretest survey, it was observed that the participants became confused when encountering the items of this construct. Thus, based on expert opinions, this section of the questionnaire was removed.

The variable of habit also deals with exploring the effect of previous use experience on accepting new technologies [26]. Since no sample of APVs has been available until conducting this study in Iran, thus it was not possible to investigate this variable and it was excluded from the APVAM model.

Regarding the price value construct, since the cost of utilizing APVs has remained unclear, the participants cannot compare the advantages gained through this technology against the costs they should pay for its usage. Thus, this variable was not directly included in the APVAM model. In some similar studies, this variable has also been altered and studied indirectly [27, 28].

In addition, other studies have highlighted two additional constructs, perceived risk (PRisk) and perceived untrustworthiness (PUnTrust), as important variables in determining the acceptance level of AVs [14, 29]. PRisk refers to the level of risk perceived by the participants toward APVs, while PUnTrust measures the lack of trust among participants. The rest of this section develops the hypotheses of the APVAM model.

Previous studies have shown that the latent variable effort expectancy (EE) has a positive and significant impact on behavioral intention (BI). This means that the more convenient is the use of AVs for users, the more likely they are to use them. In some studies, effort expectancy (EE) has been effective on behavioral intention (BI) directly [14, 22, 27, 29–32], while in some others, EE has been influential on BI indirectly through the intermediate variable of performance expectancy (PE) [8, 9, 29, 33–36]. Meanwhile, Madigan et al. concluded that the variable of effort expectancy has no effect on behavioral intention, and this construct can be removed from the UTAUT2 model [25]. These conflicting findings regarding the influence of effort expectancy on behavioral intention have led to the development of the following hypotheses:

H1—effort expectancy (EE) positively affects the performance expectancy (PE) of potential APV users.
H2—effort expectancy (EE) positively influences the behavioral intention (BI) of potential APV users.

The variable performance expectancy has consistently emerged as one of the most influential factors affecting behavioral intention in previous studies on the acceptance of AVs [8, 9, 22, 25, 29–32, 35]. This means that the more people feel that the AV is in line with their benefits, they would have the greater tendency to use them. Thus, the following hypothesis can also be propounded for APVs:

H3—performance expectancy (PE) positively affects the behavioral intention (BI) of potential APV users.

Many studies have shown that as the facilities, resources, and conditions of using technology for humans’ increase, they would have a greater tendency to use that technology. This has been introduced as a latent variable called facilitating conditions (FC) [26]. Regarding acceptance of AVs, many studies have approved the effectiveness of this variable on the degree of behavioral intention (BI) [25]. In this regard, the following hypothesis can be noted:

H4—facilitating conditions (FC) positively influence the behavioral intention (BI) of potential APV users.

As mentioned earlier, the variable of hedonic motivation (HM) means the extent of human perceived pleasure of an instrument. In studies by Venkatesh et al., it was found that this variable has a positive effect on the behavioral intention (BI) of people to buy new technology or tool [26]. Nevertheless, there is limited research exploring the impact of this variable on the behavioral intention (BI) of potential APV users. In the only study examining the effect of this relationship, a significant effect was observed [25]. Therefore, the following hypothesis can be mentioned:

H5—hedonic motivation (HM) positively affects the behavioral intention (BI) of potential APV users.

The impact of individuals’ perceived risk regarding AV technology on their behavioral intention (BI) is a subject of debate. In this regard, some studies have shown that this variable has no impact on behavioral intention (BI) [14, 37]. However, some other studies have reported conflicting results [38, 39]. Studies by Lee et al. along with Liu et al. in 2019 indicated that higher risks perceived by people about the use of AVs can negatively affect their behavior intention (BI) [38, 39]. Therefore, the following hypothesis can be stated:

H6—perceived risk (PRisk) negatively affects the behavioral intention (BI) of potential APV users.

In recent studies examining the variables affecting behavior intention (BI), new constructs have been introduced. One of these constructs is the level of perceived trust in AVs. Perceived trust is a psychological variable that impacts the acceptance of AVs. Many previous studies investigating the variable of perceived trust on behavioral intention (BI) have shown that the higher this trust to AVs, the greater the behavioral intention (BI) will be towards such vehicles. It is worth noting that in some studies, instead of measuring the level of perceived trust, the degree of perceived un-trust has been measured. According to these studies, the higher this lack of trust or un-trust, the lower the tendency of using AVs will be [9, 29, 32, 35–37, 39, 40]. In this regard, the following hypothesis has been developed:

H7—perceived Un-Trust (PUnTrust) negatively affects the behavioral intention (BI) of potential APV users.

Meanwhile, other studies have indicated that the variable of trust can influence the perceived risk negatively [14, 39, 41]. Consequently, an additional complementary hypothesis has been developed as follows:

H8—perceived un-trust (PUnTrust) can positively affect the perceived risk (PRisk) of potential APV users.

### Moderator variables

One of the issues that have remained understudied in previous studies regarding the acceptance of AVs is exploring the effect of moderator variables. In a review study, Keszey et al. inspected the studies on acceptance of AVs until 2020 and noted the lack of studies investigating the impact of moderator variables, which has hindered a clear understanding of the behavioral patterns of different groups using such vehicles [24]. While variables such as age and gender have been explored in previous studies [25, 26], this study goes beyond those variables and also examines marital status (single, married), occupation (unemployed, employed, student, retired), level of education, degree of previous familiarity with AVs, number of vehicles owned, value of the current household vehicle, value of household wealth, individualism/collectivism, type of vehicle used for daily trips (personal vehicles, public transportation, active transport modes), and the main purpose of daily trips (occupational, academic, etc.). The main objective of examining these moderator variables is to understand the differences among various groups of people in their inclination to use APVs.

The investigation of the effect of individualism and collectivism in the autonomous van acceptance model is one of the novel aspects of this research. According to numerous scholars, one of the significant characteristics of modern society is the prevalence of individualism over collectivism, resulting in changes in people’s awareness and behavior [42, 43]. In individualistic societies, the modern person finds special abilities such as rationalizing actions, breaking down traditions, an assertive selection from among new options, etc. Triandis has defined individualism as independence of opinion and reliance on rationality. He believes that individualistic people mostly rely on personal tendencies rather than current norms when determining behavior. In contrast, in collectivistic individuals, following the intragroup norms, compliance, sense of duty, accepting authority, collaboration, and conformity to a group are of high priority [43, 44].

To further explore this issue, Triandis has introduced horizontal and vertical concepts to collectivism and individualism. In horizontal individualism, the person wants to be unique and differentiated from the group, but they are not interested in bolding it, whereby that person has special characteristics such as self-belief, self-reliance, and valuing their personal goals. However, in vertical individualism, the status and position of the person are important, and to achieve this status, the person tends to compete; indeed, the main characteristics of this person are ambition, self-centeredness, and competitiveness. On the other hand, in horizontal collectivism, emphasis is put on equality in relations, i.e. collaboration, benevolence, and sense of belonging to the group. Finally, in vertical collectivism, hierarchical relations or obeying the group plus sacrifice and group integrity are of high priority [43, 44].

## Material and methods

### Questionnaire

To prepare the final questionnaire, credible questionnaires previously developed and used by researchers in this field were consulted, following typical research practices. The final questionnaire utilized in this research consists of five general sections. The first section includes a cover letter, a guideline for completing the questionnaire and an introducing to APVs, accompanied by relevant images. The second section consists of questions related to the constructs that constitute the APV acceptance model, as presented in Table 1. To prepare this section, after identifying the variables affecting the autonomous van acceptance, the questionnaire items were chosen (the items of this section were measured according to Likert scale as 1 = absolutely disagree to 5 = absolutely agree) [26]. The third section presents the items related to the individualism and collectivism scale (the items of this section were measured with the range of 1 = never or absolutely not to 5 = always or definitely yes). The items related to the acceptance of APVs for individualism and collectivism scale are provided in Table 1. The fourth section collects information regarding the participants’ daily intracity travel habits, including the type of vehicle they use and their purpose for these trips. Finally, the fifth section gathers demographic information from the participants, such as age, place of residence, gender, marital status, level of education, and economic status.

**Table 1:** Constructs, their items, and sources.

Constructs	Items	Mean	SD	Source adapted
**Behavioral intentions**	BI1: Assuming that I had access to an APV, I predict that I would use it in the future.	4.15	0.751	Madigan et al., 2017
	BI2: If an APV becomes available permanently, I plan to use it.	4.01	0.810	
**Performance expectancy (Perceived Usefulness)**	PE1: I find the APV a useful mode of transport.	4.08	0.875	Madigan et al., 2017
	PE2: Using the APV to travel helps me to achieve things that are important to me.	3.63	0.914	
	PE3: Using the APV will help me reach my destination more quickly.	3.48	0.970	
**Effort expectancy (Perceived Ease of Use)**	EE1: My interaction with the APV is clear and understandable.	3.55	0.948	Madigan et al., 2017
	EE2: I find the APV easy to use.	3.64	0.913	
	EE3: Learning to use an APV is easy for me.	4.15	0.769	
**Facilitating conditions**	FC1: I have the resources necessary to use an APV.	3.87	0.916	Madigan et al., 2017
	FC2: I know necessary to use an APV.	3.54	1.030	
	FC3: The APV is compatible with other forms of transport I use.	3.90	0.864	
	FC4: I can get help from others when I have difficulties using an APV.	3.52	0.954	
**Hedonic motivation**	HM1: Using an APV is fun (enjoyable).	4.18	0.861	Madigan et al., 2017
	HM2: Using an APV is entertaining.	3.99	0.868	
**Perceived (Un) Trust (PT)**	PT1: I generally have concerns about using an APV.	3.51	1.025	Panagiotopoulos & Dimitrakopoulos, 2018
	PT2: APV is somewhat frightening to me.	2.74	1.052	
	PT3: I have concerns about the safety of the APV.	3.56	1.026	
	PT4: I have concerns about the system security and data privacy of the APV.	3.33	1.088	
	PT5: I am against the use of APVs by my family.	2.57	1.043	Researcher made
**Perceived risk**	PR1: Using an APV would be risky.	2.69	0.972	Choi & Ji, 2015
	PR2: APV might not perform well and create problems.	3.08	0.977	
	PR3: APV would lead to a financial loss for me.	2.53	0.856	
**Horizontal individualism items**	HI1: I’d rather depend on myself than others.	4.05	1.100	Triandis, & Gelfland, 1998
	HI2:I rely on myself most of the time; I rarely rely on others.	4.01	0.930	
	HI3:I often do "my own thing."	4.12	0.810	
	HI4:My personal identity, independent of others, is very important to me.	4.23	0.884	
**Vertical individualism items**	VI1: It is important that I do my job better than others.	4.23	0.925	Triandis, & Gelfland, 1998
	VI2: Winning is everything.	3.21	1.201	
	VI3: Competition is the law of nature.	3.70	1.124	
	VI4: When another person does better than I do, I get tense and aroused.	2.61	1.223	
**Horizontal collectivism items**	HC1: If a coworker gets a prize, I would feel proud.	3.45	1.078	Triandis, & Gelfland, 1998
	HC2: The well-being of my coworkers is important to me.	3.82	1.000	
	HC3: To me, pleasure is spending time with others.	3.30	1.153	
	HC4: I feel good when I cooperate with others.	3.94	0.951	
**Vertical collectivism items**	VC1: Parents and children must stay together as much as possible.	3.47	1.153	Triandis, & Gelfland, 1998
	VC2: I must take care of my family, even when I have to sacrifice what I want.	3.65	1.158	
	VC3: Family members should stick together, no matter what sacrifices are required.	3.41	1.147	
	VC4: It is important to me that I respect the decisions made by my groups.	3.90	0.923	

After preparing the initial version of the questionnaire, a pretest survey was conducted. The questionnaire was administered to 15 participants, including both university professors and individuals from the general population, in Persian language to ensure smooth translation. Participants provided feedback and suggestions, leading to adjustments in vocabulary choices and merging of certain items. Based on these recommendations, the questionnaire was modified accordingly. Eventually, the online version of the questionnaire was created using the Porsall.com platform.

### Participants

In this research, a total of 824 Tehran citizens participated by completing questionnaires distributed online through the Internet and social media platforms. Among the respondents, 462 were male (56.1%) and 318 were female (38.6%), and 44 participants (5.3%) did not provide a response regarding their gender. Further, 626 (76%) of participants in the survey announced themselves as single.

In terms of education, the breakdown was as follows: below diploma (3%), diploma (11.2%), bachelor’s degree (47.5%), master’s degree or graduate professional (34.7%), and specialized PhD or medical specialist degrees (3.1%).

The participants had an average age of 25.5 (SD = 7.27) years. 3.1 percent of the participants were aged under 18 years, 28.5% were aged 19–22 years, 56.3% were aged 23–30 years, 10% were aged 31–50 years, and 2.1% were aged 51 years and above. It is important to note that the findings of this study are limited to young citizens of Tehran, and generalization to other age groups should be done cautiously.

To ensure a representative sample, efforts were made to select participants from each of the 22 districts of Tehran in proportion to the population distribution. Pearson correlation analysis was conducted to examine the relationship between the number of participants from each district and the population residing in those districts [45]. The correlation coefficient was found to be 0.981, indicating a significant correlation at the 0.01 level.

### Survey procedure

Before conducting the study, ethical approval was granted by the department of transportation engineering and planning of the Sharif University of technology ethics committee. The research panel sent invitations of the survey to different channels and groups across social media such as Telegram, WhatsApp, and Instagram, and invited people to participate in the study. All participants were the research panel’s voluntary respondents and they were assured that their responses would be both confidential and anonymous. Also, the participants have declared their written consent to participate in this study and have confirmed the following statement by clicking the final registration button on the online questionnaire: "I consent to participate in this research project and the following has been explained to me: my participation is completely voluntary. my right to withdraw from the study at any time without any implications to me." The conversion rate was approximately 5%. The questionnaire link for this research was active from November 1, 2019, to January 10, 2020, allowing participants to respond to the questionnaire items at their convenience.

### Data analysis

In this research, after preprocessing the data and resolving problems such as missing data, the relationships between the variables affecting the acceptance of APVs were investigated using structural equations modeling. In the data preprocessing stage, missing values for all variables were replaced with their median value, except for the variable of age, where missing values were replaced with the mean age of the participants.

In the 1970s, a revolution occurred in the application of structural equation modeling, especially in sociology and economics. Unlike other multivariate analysis methods, structural equation modeling deals with the concurrent investigation of a series of interrelations; indeed, they can be considered as a set of multiple regression equations. A structural equations model consists of structural patterns and measurement patterns. This structural pattern refers to a set of interdependencies that interrelate the constructs in the pattern. On the other hand, the measurement pattern is part of a general model which specifies the measurement indices related to each latent variable that cannot be measured directly, rather it should be measured by some other variables (indices).

There are several approaches to SEM, including Covariance-based SEM (CB-SEM), Partial Least Squares (PLS), Generalized Structural Component Analysis (GSCA), and Nonlinear Universal Structural Relational Modeling (NEUSREL) [46]. In covariance-based SEM (CB-SEM), large data with normal distribution are required, and since this cannot always be achieved, researchers have found a tendency toward using partial least squares (PLS) [46]. Briefly, it can be stated that the PLS is a suitable alternative to CB-SEM when the sample size is small, the available theory is limited, and that prediction accuracy is important [47, 48]. In this research, SmartPLS v3.2.8 was utilized for modeling the structural equations [49].

## Results

### Descriptive results

Table 2 presents the descriptive statistics pertaining to the participants in this research. Only 25.7% of the participants in this research stated that they have never heard even the name of AVs so far, and all of their information about these vehicles was related to the vehicle introduction section in the survey. Furthermore, over 85% of participants had an academic level of education. Also, 92.2% of the participants were those younger than 35 years. The results of the individualism/collectivism questionnaire indicated that 524 participants (63.6%) displayed individualistic tendencies, while300 participants (36.4%) exhibited collectivist tendencies.

**Table 2:** Descriptive statistics.

Variable/Items	Frequency	Percent (%)	Valid percent (%)
**AV Familiarity** (Before studying the text of introducing APVs in this questionnaire, how much had you heard about such vehicles?)			
I had not heard even its name	212	25.7	25.7
I had heard only its name	235	28.5	28.5
I have followed their news to some extent and was familiar with their notion	280	34.0	34.0
I well knew about them.	97	11.8	11.8
Missing value	0	0.0	-
**Daily Travel Data** (What modes of transport do you mostly use for your daily travel?)			
Personal vehicle (personal transport)	215	26.1	27.5
Motorcycle (personal transport)	15	1.8	1.9
Intracity taxi (public transport)	81	9.8	10.4
Intracity bus (fixed-route or Bus Rapid Transport) (public transport)	137	16.6	17.5
Subway (public transport)	235	28.5	30.1
Bicycle (active transport mode)	5	0.6	0.6
Walking (active transport mode)	42	5.1	5.4
Internet taxi (personal vehicle)	48	5.8	6.1
Phone taxi agencies (personal vehicle)	4	0.5	0.5
Missing value	42	5.1	-
**Trip Purpose** (What is the main purpose of your daily trips?)			
Occupational	225	27.3	28.8
Academic	423	51.3	54.1
Shopping	50	6.1	6.4
Entertainment	55	6.7	7.0
Carrying family members	19	2.3	2.4
Other	10	1.2	1.3
Missing value	42	5.1	-
**Gender**			
Female	318	38.6	40.8
Male	462	56.1	59.2
Missing value	44	5.3	-
**Marriage States**			
Married	154	18.7	19.7
Unmarried	626	76.0	80.3
Missing value	44	5.3	-
**Education Level**			
Below diploma	23	2.8	3.0
Diploma and associate degree	92	11.2	11.8
Bachelor’s	37	44.9	47.5
MSc / MA / GP	270	32.8	34.7
PhD	24	2.9	3.1
Missing value	45	5.5	-
**Household Car Ownership** (How many cars are there in your household?)			
Zero Car	60	7.3	7.7
One Car	398	48.3	51.1
Two Car	229	27.8	29.4
Three Car	70	8.5	9.0
More than Three Car	22	2.7	2.8
Missing value	45	5.5	-
**Residence Ownership Situation** (Type of ownership of the residence?)			
Owner	372	45.1	47.8
Tenant	175	21.1	22.5
Living with parents	232	28.2	29.8
Missing value	45	5.5	-
**Residence Area** (What is the residential area in terms of the meter?)			
Below 60 m^2^	56	6.8	7.2
60–80 m^2^	109	13.2	14.1
80–100 m^2^	146	17.7	18.9
100–120 m^2^	165	20.0	21.3
120–150 m^2^	159	19.3	20.6
Larger than 150 m^2^	138	16.7	17.9
Missing value	51	6.2	-
**Car Price** (If there is a personal car in your household, what is the approximate range of its price? (If there is more than one car, consider the most expensive one))			
We have no personal vehicle	49	5.9	6.3
Less than 500 million Rials ($4000)	156	18.9	20.2
Between 500 million and 1 billion Rials ($4000–8000)	282	34.2	36.5
1–1.5 billion Rials ($8000–12000)	109	13.2	14.1
1.5–2 billion Rials ($12000–16000)	51	6.2	6.6
2–2.5 billion Rials ($16000–20000)	32	3.9	4.1
2.5–3 billion Rials ($20000–24000)	27	3.3	3.5
3–5 billion Rials ($24000–40000)	34	4.1	4.4
5–10 billion Rials ($40000–80000)	28	3.4	3.6
Larger than 10 billion Rials ($>80000)	5	0.6	0.6
Missing value	51	6.2	-
**Job Situation** (What is your occupational status?)			
Full-time Employed	146	17.7	18.9
Part-time Employed	143	17.4	18.5
Unemployed	100	12.1	12.9
Retired	21	2.5	2.7
Full-time student	293	35.6	37.9
Part-time Student	70	8.5	9.1
Missing value	51	6.2	-
**Household Wealth**			
Level 1- The first to the third tenth economic level (Less than 84000$)	275	33.3	33.3
Level 2- The fourth to the seventh tenth economic level (84000$ - 185000$)	274	33.3	33.3
Level 3- The eighth First to the ten tenth economic level (More than 185000$)	275	33.3	33.3

One of the important variables of socioeconomic characteristics of people which can affect the degree of tendency to using new technologies such as AVs is the monthly household income. However, considering the characteristics of Iranian people, who are often reluctant to disclose their actual income, no questions were included in this study regarding this matter. Instead, a new variable called "household wealth" was developed to investigate the financial status of the participants’ families. This variable was derived from other information collected, such as the number of household vehicles, the price range of these vehicles, and the type of residence. These factors were used to approximate the financial level of the participants’ families. The value of the household wealth variable was calculated according to Formula 1.

HouseholdWealth=HouseValue+CarValue (1)

$$House\;Value=\left\{ \begin{array}{c} House\;Area\times Mean\;Price\;of\;Apartment\;in\;i^{'}th\;District(per\;meter)\\ \;if\;HouseOwnership=owner\\ \frac{1}{5}(House\;Area\times Mean\;Price\;of\;Apartment\;in\;i^{'}thDistrict(per\;meter))\\ \;if\;HouseOwnership=tenant \end{array}\right.$$

$$Car\;Value=\left\{ \begin{array}{l} 0\;if\;Car\;No.=0\\ Car\;Price\;if\;Car\;No.=1\\ 1.5\times Car\;Price\;if\;Car\;No.=2\\ 2\times Car\;Price\;if\;Car\;No.=3\\ 3\times Car\;Price\;if\;Car\;No.=more\;than\;3 \end{array}\right.$$

As shown in Formula 1, household wealth is equal to the total Rial value (Iran’s Currency) of the house of residence and the household vehicles. To calculate the Rial value of the place of residence of the participant, the mean price of each meter of an apartment at the time of sampling was extracted for every 22 districts of Tehran according to the report published by the Ministry of Road and Urban Development of Iran (MRUD). Next, in case the participant had reported that they live in their own house, the value of their place of residence has been calculated according to the above relations. For those who said they live in a rented house since the value paid for a complete mortgage of an apartment in Tehran at the time of sampling has been about 1/5 of the value of the house on average, the value of the place of residence of the respondent was approximated as 1/5 of the total value of the apartment their family lived in. Note that since the participant was asked to report the price range of their most expensive vehicle owned by the family, thus according to the above formulas, by applying a reduction coefficient in the number of the household vehicles, the value of their vehicle has been estimated. The output results of this variable indicated that the mean household wealth of the participants in this study has been 1112.0 ($8896) - 79943.4 ($639547.2) million Rials. The mean calculated for the variable of household wealth has been 18290.5 million Rials ($146324) with the standard deviation of 12827.8 million Rials ($102622.4).

### Model assessment

**Fig 2:**
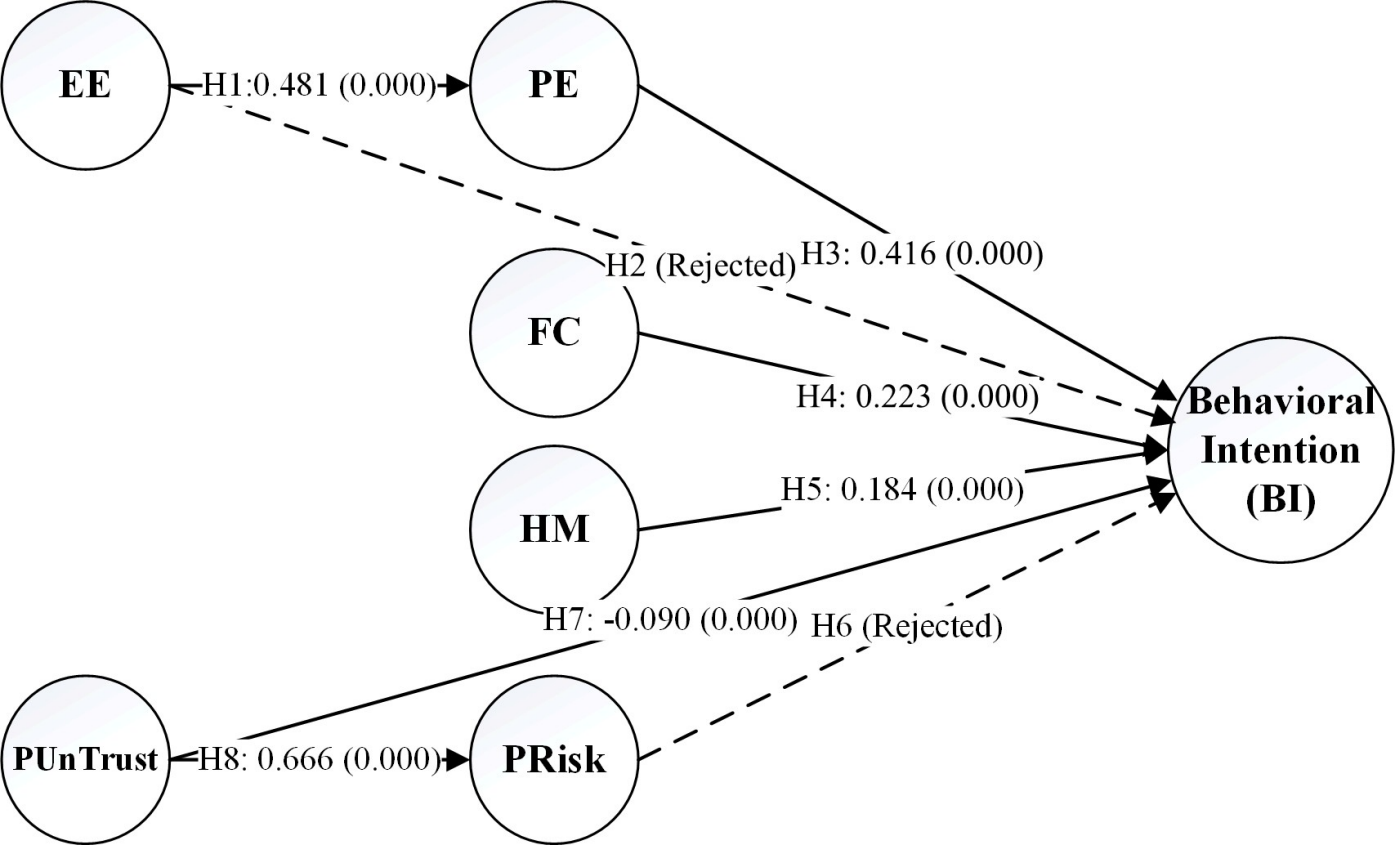
The results of SEM and final APVAM. The numbers on the diagram indicate path coefficients, and those inside the parenthesis are p-value.

The proposed APVAM model was modeled in SmartPLS software. The values of path coefficient (β) for H2 and H6 hypotheses were 0.036 (t-value = 1.072, p-value = 0.291) and -0.008 (t-value = 0.231, p-value = 0.838). The modeling results indicated that the path coefficient values and significance levels did not support the H2 and H6 hypotheses. Fig 2 presents the results of structural equations modeling and the final APVAM.

Structural equation modeling through partial least square (PLS_SEM) lacks a total estimation index for processing the models developed by this method. However, to evaluate the models developed with PLS, various criteria and indices have been presented for evaluating the measurement models and structural models in PLS literature [50]. The rest of this section presents the results of the assessment of the final APVAM at both measurement and structural modeling levels.

#### Measurement model assessment

All measurement models utilized in APVAM have been of the reflective model type. A reflective measurement model refers to a model in which the observable or explicit variable reflects its counterpart latent variable, where every observable represents an error-prone measurement of the latent variable. The reflective measurement model test is categorized into three groups of reliability, validity, and overall quality of the measurement model.

In reliability tests of the reflective measurement model, the homogeneity and uni-dimensionality conditions of each construct are investigated. For this purpose, three indices have been introduced including the reliability of each observable variable, Cronbach alpha, and composite reliability (CR). To explore the reliability of observable variables, the absolute value of the factorial load of each observable variable should be at least 0.7. However, some studies have suggested that that variable would be eliminated only if its factorial load value is less than 0.4; otherwise, that variable is suggested to be kept [51]. Cronbach’s alpha is a traditional index used to assess the reliability of observable variables in a reflective measurement model, with a value of 0.7 considered acceptable for this index [52]. As mentioned earlier, the next criterion for evaluating the reliability of the measurement models is composite reliability, whose values larger than 0.7 indicate the internal consistency of the measurement model [53].

The validity test of the reflective measurement model is investigated at two levels of convergent validity and discriminant validity. Convergent validity refers to measuring the extent of interpreting the latent variable by its observable variables. To measure this index, the average variance extracted (AVE) criterion is used. The minimum acceptable value for this index is 0.5, meaning that the desired latent variable interprets at least 50% of the variance of its observable variables. Discriminant validity measures the ability of a reflective measurement model in the extent of differentiating observables of the latent variable from that model with other observables in the model. To measure this index, the Fornell-Larker test has been introduced. According to this test, the mean AVE of each latent variable should be larger than the second power of the correlation of that latent variable with other latent variables [54].

The final test for the reflective measurement model is the quality test, which is measured by construct cross-validated community (CVCom). If this index indicates a positive number, then the measurement model has the required quality.

Table 3 presents the results related to these tests for the reflective measurement model, while Table 4 provides the results of the Fornell-Larker test. As the results show, all of the tests fall within an acceptable range. Thus, it can be concluded that the reflective measurement models in the final APVAM model exhibit acceptable reliability, validity, and quality.

**Table 3:** The results related to the reliability, validity, and quality tests for the APVAM measurement model.

Constructs	Indicator	Factor loading	t-value	p-value	Cronbach’s alpha	CR	AVE	CV Com
**Behavioral intentions (BI)**	BI1	0.891	76.600	0.000	0.7508	0.8892	0.8005	0.3447
	BI2	0.899	102.688	0.000				
**Performance expectancy (PE)**	PE1	0.824	62.113	0.000	0.7919	0.8772	0.7045	0.3878
	PE2	0.878	79.076	0.000				
	PE3	0.814	51.365	0.000				
**Effort expectancy (EE)**	EE1	0.836	52.667	0.000	0.7417	0.8537	0.6619	0.3301
	EE2	0.876	74.456	0.000				
	EE3	0.721	25.383	0.000				
**Facilitating conditions (FC)**	FC1	0.830	55.351	0.000	0.6994~0.70	0.8130	0.5260	0.2438
	FC2	0.719	26.342	0.000				
	FC3	0.770	41.091	0.000				
	FC4	0.552	12.889	0.000				
**Hedonic motivation (HM)**	HM1	0.921	131.492	0.000	0.7489	0.8868	0.7968	0.3377
	HM2	0.863	47.997	0.000				
**Perceived UnTrust (PUnTrust)**	PT1	0.666	21.952	0.000	0.7380	0.8256	0.4899~0.5	0.2595
	PT2	0.818	75.175	0.000				
	PT3	0.754	37.861	0.000				
	PT4	0.573	17.131	0.000				
	PT5	0.664	22.759	0.000				
**Perceived risk (PR)**	PR1	0.810	46.487	0.000	0.7745	0.8683	0.6877	0.3675
	PR2	0.795	42.647	0.000				
	PR3	0.880	109.400	0.000				

**Table 4:** Results of Fornell-Larker test for assessing the measurement model.

Constructs	(BI)	(FC)	(HM)	(EE)	(PR)	(PUnTrust)	(PE)
**(BI)**	**0.8947**	*	*	*	*	*	*
**(FC)**	0.5559	**0.7253**	*	*	*	*	*
**(HM)**	0.4947	0.4450	**0.8926**	*	*	*	*
**(EE)**	0.4450	0.6309	0.3339	**0.8136**	*	*	*
**(PR)**	-0.3470	-0.3911	-0.2692	-0.3416	**0.8293**	*	*
**(PUnTrust)**	-0.2711	-0.2829	-0.1647	-0.2721	0.6658	**0.7000**	*
**(PE)**	0.6429	0.5425	0.4725	0.4814	-0.3541	-0.2110	**0.8394**

#### Structural model assessment

In the structural model, the relations between latent variables (including both dependent and independent) are of interest. The criteria for testing the structural model include: 1- the index of coefficient of determination (R^2^) for endogenous latent variables, 2- path coefficients (β) and their significance, 3- the Stone-Geisser’s Q^2^ value (Q^2^), and 4- Cohen effect size criterion (f^2^) [53, 55].

One of the most important assessment criteria for endogenous latent variables in the path model is coefficient of determination or R^2^. Values of 0.19, 0.33, and 0.67 for R^2^ are considered weak, average, and substantial, respectively. The value of the coefficient of determination for exogenous variables (independent) is 0 [53]. In the second column of Table 5, R^2^ values calculated for the endogenous latent variables have been provided. As the results show, the values of coefficient of determination for BI and PE variables have been average to considerable, while for PR it has been weak to average.

**Table 5:** R^2^ and Q^2^ results.

Constructs	R^2^	Q^2^
**Behavioral intentions (BI)**	0.5068	0.3834
**Performance expectancy (PE)**	0.4433	0.1528
**Perceived risk (PR)**	0.2317	0.2841

The next criterion for investigating a structural model is path coefficients, also known as the standardized beta in linear regression. Path coefficients are important in terms of magnitude, significance, and sign. A positive path coefficient means a positive relationship, while a negative coefficient indicates an inverse relationship between endogenous and exogenous latent variables. The significance of path coefficients is determined by the t-value, with thresholds of 1.64, 1.96, and 2.58 corresponding to significance levels of 90%, 95%, and 99% respectively [53, 56]. Table 6 present the values of path coefficient plus t-value and p-value. In the final model, all path coefficients are found to be significant at the 99% level.

**Table 6:** Path coefficient of APVAM, t-value and p-value.

Path (Hypothesis)	Path Coefficient (β)	t-value (p-value)	f^2^	Result
**H1: EE→PE**	0.481	15.279 (0.000)	0.3016	Confirmed
**H2: EE→BI**	0.036	1.072 (0.291)	-	Rejected
**H3: PE→BI**	0.416	13.188 (0.000)	0.2235	Confirmed
**H4: FC→BI**	0.223	6.855 (0.000)	0.0639	Confirmed
**H5: HM→BI**	0.184	5.474 (0.000)	0.0499	Confirmed
**H6: PR→BI**	-0.008	0.231(0.838)	-	Rejected
**H7: PUnTrust →BI**	-0.090	3.805 (0.000)	0.0150~0.02	Confirmed
**H8: PUnTrust →PR**	0.666	26.673 (0.000)	0.7962	Confirmed

Stone-Geisser’s Q^2^ value is another index in evaluating structural models and their quality, dealing with investigating the ability of the structural model in prediction [57, 58]. Q^2^ values larger than zero indicate that the observed values have been well represented and the structural model has a good prediction ability. Values of 0.02, 0.15, and 0.35 represent weak, Average, and strong, respectively [53].

Eventually, the last criterion for investigating the structural model is Cohen’s size effect criterion (f^2^). This criterion measures the effect size of an exogenous variable on an endogenous variable in a structural model. Cohen for this criterion has introduced 0.02, 0.15, and 0.35 for the effect sizes of weak, average, and strong, respectively [55]. Also, f^2^ values have been provided in the fourth column of Table 6. The results show that the variable of performance expectancy has the largest impact on the behavioral intentions of the potential users of APVs among all the variables.

### Moderator variables effect analysis

Various methods have been introduced in the structural equations modeling literature to calculate the effect size of moderator variables. Among these methods, the stage approach [59] and the product indicator approach [60] are widely used for moderator variable analysis. If the moderator variable and exogenous variable in the structural model are of the interval or continuous type, and their measurement model is of a reflective type, the product indicator approach is used for calculating the moderating level of them. On the other hand, if one of the two variables is a moderator or exogenous qualitative, or if the measurement model is a formative type, a staged approach is employed.

#### Stage approach results

As explained in the conceptual research model and hypotheses development section, the moderating effect of 12 moderator variables has been explored on the APVAM. Since six studied variables including individualism/collectivism, gender, marital status, occupation, highly used mode of transport, and aims of trip are qualitative, to investigate the moderating level of these variables, a stage approach was used. The results obtained from the stage approach in examining the moderating effects of these six variables are presented in Table 7.

**Table 7:** The results of investigating the moderating variables studied by stage approach.

Moderator variable		FC → BI	HM→BI	EE→PE	PUnTrust →BI	PUnTrust → PRisk	PE→BI
**Collectivism vs Individualism**	Path Coefficients (COLC)	0.2501	0.0973	0.4955	-0.0497	0.69	0.4782
	Path Coefficients (INDV)	0.2176	0.2313	0.4746	-0.1144	0.6558	0.3804
	t-Value (COLC vs INDV)	0.5194	**2.0672** ^**^	0.3294	1.2747	0.6989	1.5242
**Gender**	Path Coefficients (FEMALE)	0.1734	0.2449	0.5252	-0.1175	0.7207	0.3877
	Path Coefficients (MALE)	0.2572	0.1441	0.4569	-0.0726	0.6305	0.4282
	t-Value(MALE vs FEMALE)	1.301	1.5785	1.0767	0.885	**1.797** ^*^	0.6421
**Marriage states**	Path Coefficients (Married)	0.1223	0.2548	0.6409	-0.0613	0.692	0.4594
	Path Coefficients (Single)	0.2495	0.1632	0.4451	-0.0993	0.6637	0.4039
	t-Value(Married vs Single)	**1.6713** ^*^	1.1323	**2.6154** ^**^	0.5965	0.457	0.6995
**Job States**	Path Coefficients (Job: Retired)	0.2635	0.5807	0.6745	0.0148	0.8705	0.1908
	Path Coefficients (Job: Student)	0.2178	0.1642	0.3831	-0.1151	0.6785	0.4039
	Path Coefficients (Job: Unemployed)	0.1417	0.1461	0.5416	-0.0303	0.7358	0.5125
	Path Coefficients (Job: employed)	0.2509	0.1945	0.5247	-0.0932	0.6056	0.3922
	t-Value(Job: Retired vs Job: Student)	0.2375	**1.9304** ^*^	1.586	0.7544	1.1444	1.1609
	t-Value(Job: Retired vs Job: Unemployed)	0.633	**2.3224** ^**^	0.6684	0.3009	1.3965	1.5721
	t-Value(Job: Retired vs Job: employed)	0.0584	**2.0169** ^**^	0.8018	0.769	**1.7147** ^*^	0.8946
	t-Value(Job: Student vs Job: Unemployed)	0.9135	0.2023	**1.9178** ^*^	1.1839	0.8616	1.3649
	t-Value(Job: Student vs Job: employed)	0.4579	0.4196	**2.1376** ^**^	0.391	1.2497	0.1655
	t-Value(Job: Unemployed vs Job: employed)	1.1661	0.5792	0.1939	0.9928	**2.0706** ^**^	1.26
**Travel Mode**	Path Coefficients (Travel Mode: Active)	0.2264	-0.0582	0.5704	-0.1421	0.834	0.4598
	Path Coefficients (Travel Mode: private)	0.1436	0.1999	0.5608	-0.0693	0.6196	0.4831
	Path Coefficients (Travel Mode: public)	0.245	0.2052	0.4229	-0.0999	0.6798	0.3788
	t-Value(Travel Mode: Active vs Travel Mode: private)	0.6175	**1.8969** ^*^	0.0745	0.6386	**1.7958** ^*^	0.1629
	t-Value(Travel Mode: Active vs Travel Mode: public)	0.1356	**1.8111** ^*^	1.0715	0.3736	**1.7277** ^*^	0.6047
	t-Value(Travel Mode: private vs Travel Mode: public)	1.5219	0.0784	**2.118** ^**^	0.5868	1.1738	1.5793
**Travel Purpose**	Path Coefficients (Travel Purpose: Education)	0.2246	0.1655	0.4315	-0.0587	0.6814	0.4231
	Path Coefficients (Travel Purpose: Else)	0.1669	0.2394	0.6135	-0.0878	0.5936	0.4355
	Path Coefficients (Travel Purpose: job)	0.2541	0.1814	0.4958	-0.1337	0.7007	0.3882
	t-Value(Travel Purpose: Education vs Travel Purpose: Else)	0.6719	0.8019	**2.2192** ^**^	0.4058	1.3149	0.1509
	t-Value(Travel Purpose: Education vs Travel Purpose: job)	0.4144	0.2131	0.9149	1.2457	0.3973	0.4983
	t-Value(Travel Purpose: Else vs Travel Purpose: job)	0.9264	0.689	1.4802	0.6412	1.3799	0.4698

* Path coefficients difference which is significant at p < 0.1 level.

** Path coefficients difference which is significant at p < 0.05 level.

The results of examining the individualism/collectivism variable with the stage approach indicated that this variable had moderating effect only on the HM to BI path coefficient. This indicates that for individualistic people (β = 0.2313), as compared to collectivist individuals (β = 0.0973), the fun and pleasure they perceive when using APVs is an important factor in their decision for utilizing the APVs. However, being individualistic or collectivist had no moderating effect on other paths of the APVAM, and the results were consistent for both individualistic and collectivist individuals.

By investigating the results obtained from moderating effect of the gender variable, it was found that this variable has moderating effects only in the PUnTrust to PRisk path, while gender has no moderating effect in any other part of the APVAM. According to the results, women PUnTrust has had a greater impact on their perceived risk for using APVs compared to men.

The variable of marital status had a moderating effect on FC → BI and EE → PE paths. Analysis results showed that for single individuals (β = 0.2495), the level of resources and support of APVs is more important for them to use this technology (FC) compared to married individuals (β = 0.1223). The results indicated that the marital status variable had no impact in other parts of the APVAM.

The next variable investigated was occupation. Four occupational states were explored including employed, student, unemployed, and retired. The results of the stage approach analysis showed that the HM → BI path was more important for retired people (β = 0.5807) compared to employed (β = 0.1945), students (β = 0.1642), and unemployed (β = 0.1461). This means that fun and pleasure are of more importance for the retired people compared to others. The EE → PE path coefficient for students (β = 0.3831) was less important compared to the employed (β = 0.5416) and unemployed (β = 0.5416) people. This means that the convenience of using the technology is less important for students compared to both employed and unemployed people. Eventually, for the PUnTrust → PRisk path coefficient, the results showed that the employed individuals (β = 0.6056) had lower values compared to unemployed (β = 0.7358) and retired people (β = 0.8705).

Then, three general states were investigated for the variable of a widely used mode of transport including private transport (private vehicle, private motorcycle, Internet taxi, and telephone taxi), public transport (town car, bus, and subway), and active transport mode (bicycle and walking) as moderating variable. The results of stage approach analysis showed that those who currently benefit from active transport mode for their intracity trips showed a negative moderating effect on the HM → BI path. This means that perceived fun and pleasure for APVs have a reductive effect on the tendency to use such vans for the individuals who employ active transport mode (β = -0.0582). This has been opposite significantly for the individuals who use private (β = 0.1999) or public (β = 0.2052) transport for their intracity trips. On the other hand, in the PUnTrust → PRisk path, the individuals who use active transport mode (β = 0.8340) for their daily trips showed higher PUnTrust causing more perceived risk in them compared with individuals who use private (β = 0.6196) or public (β = 0.6798) transport more.

The last moderator variable examined via the stage approach in this study is the purpose of the trip variable including occupational, academic, and others. This means that the participants were asked to say their main purpose of intracity daily trips. The results showed that the only moderating effect of this variable occurred in EE → PE path between occupational (β = 0.4958) and academic (β = 0.4315) trips. This analysis suggests that for individuals whose daily intracity trips are mostly occupational, the level of ease in using APVs has a greater impact on their perception of its efficiency compared to those whose trips are primarily academic-oriented.

#### Product indicator approach results

Since six variables of age, level of education, the degree of the previous familiarity with APVs (AV familiarity), the number of household vehicles, price of household vehicles, and the household wealth are of discrete or continuous type, the product indicator approach was used to investigate the moderating level of these variables in the APVAM path coefficients. The results obtained from this approach in exploring the moderating effect of these six variables are presented in Table 8.

**Table 8:** The results of examining the moderating variables studied via the product indicator approach.

Moderator Variable		FC → BI	HM→BI	EE→PE	PUnTrust →BI	PUnTrust → PRisk	PE→BI
**AGE**	Path Coefficient	**0.073** ^*^	0.008	0.011	-0.047	0.2	0.027
	t-value	**1.637~1.64** ^*^	0.216	0.313	1.316	0.557	1.004
	p-value	**0.102~0.1** ^*^	0.829	0.755	0.189	0.578	0.316
**Education Level**	Path Coefficient	0.009	**0.043** ^**^	-0.035	-0.01	-0.046	0.032
	t-value	0.416	**2.049** ^**^	1.278	0.483	1.583	1.223
	p-value	0.667	**0.041** ^**^	0.202	0.629	0.114	0.222
**AV Familiarity**	Path Coefficient	-0.013	-0.003	**-0.078** ^**^	0.016	-0.011	-0.004
	t-value	0.518	0.086	**2.689** ^**^	0.701	0.339	0.171
	p-value	0.604	0.932	**0.007** ^**^	0.484	0.735	0.864
**Car no.**	Path Coefficient	**-0.046** ^**^	0.026	0.039	0.046	0.025	-0.003
	t-value	**2.036** ^**^	1.016	1.164	1.787	0.849	0.101
	p-value	**0.042** ^**^	0.31	0.245	0.074	0.397	0.919
**Car Price**	Path Coefficient	-0.023	**0.064** ^**^	0.059	0.052	-0.005	0.035
	t-value	0.761	**2.346** ^**^	1.871	1.925	0.18	1.166
	p-value	0.447	**0.019** ^**^	0.062	0.055	0.857	0.244
**Household Wealth**	Path Coefficient	-0.019	0.03	0.05	0.025	0.001	0.033
	t-value	0.704	1.243	1.598	0.901	0.024	1.034
	p-value	0.482	0.214	0.111	0.368	0.981	0.302

* Path coefficients which are significant at p < 0.1 level.

** Path coefficients which are significant at p < 0.05 level.

The results of investigating the variable of age via product indicator approach showed that this variable has had moderating effect only in the FC → BI path of APVAM. This means that for older individuals, strong and efficient support of AVs is more effective on their tendency to using APVs compared to younger people.

By investigating the results obtained from the moderating effect of the variable of the level of education, it was found that this variable had a moderating effect only on the HM → BI path of APVAM. According to the results, as the level of education of participants increases, the fun and pleasure perceived by the people about APVs would have a greater impact on their tendency to utilize such vehicles.

One of the primary questions asked to the participants after studying the introduction section and the questionnaire guidelines was their level of previous familiarity with APVs. The results of this question were reported under a variable called AV familiarity. The results showed that this variable had moderating effect only on the EE → PE path of APVAM. This means that with higher previous familiarity with APVs, the extent of ease of use of APVs was associated with less impact on the degree of fulfilling the people’s benefits in line with their needs. As a possible explanation, it can be stated that media coverage about AVs has indirectly influenced people’s inclination to use such vehicles in a negative way.

The next variable investigated by the product indicator approach was the variable of the household car number (Car No.). The results showed that this variable has a moderating effect only on the FC → BI path. According to the results, as the number of cars owned by the family increases, the importance of support for APVs decreases in determining the extent of people’s tendency to applying these vehicles.

The variable of car price indicates the price of the most expensive car owned by the participants’ households. The results of investigating the moderating effect of this variable showed that the more expensive this vehicle, the more important is the HM → BI path coefficient. This means that for individuals who currently own a more expensive car, the perceived pleasure and fun for AVs is a more effective factor in increasing their tendency to using these vehicles compared to the individuals who currently own less expensive cars.

The last variable investigated using the product indicator approach was household wealth. The results showed that this variable has no significant moderating effect on any of the APVAM paths. This may be due to the fact that since APVs are categorized as public transport with a fixed usage price, the difference in household wealth did not have a significant impact on the results across various groups.

One of the important results of investigating the moderating variables whether through stage or product indicator approach, is that none of the moderating variables had a moderating effect on PE → BI and PUnTrust → BI paths.

## Discussion

The results of descriptive statistical analysis showed that the participants in this study have a greater tendency to utilizing APVs (BI Mean = 4.08). This suggests a high tendency of Tehran citizens to using the newly emerged technology of APVs as a mode of public transport.

**Fig 3:**
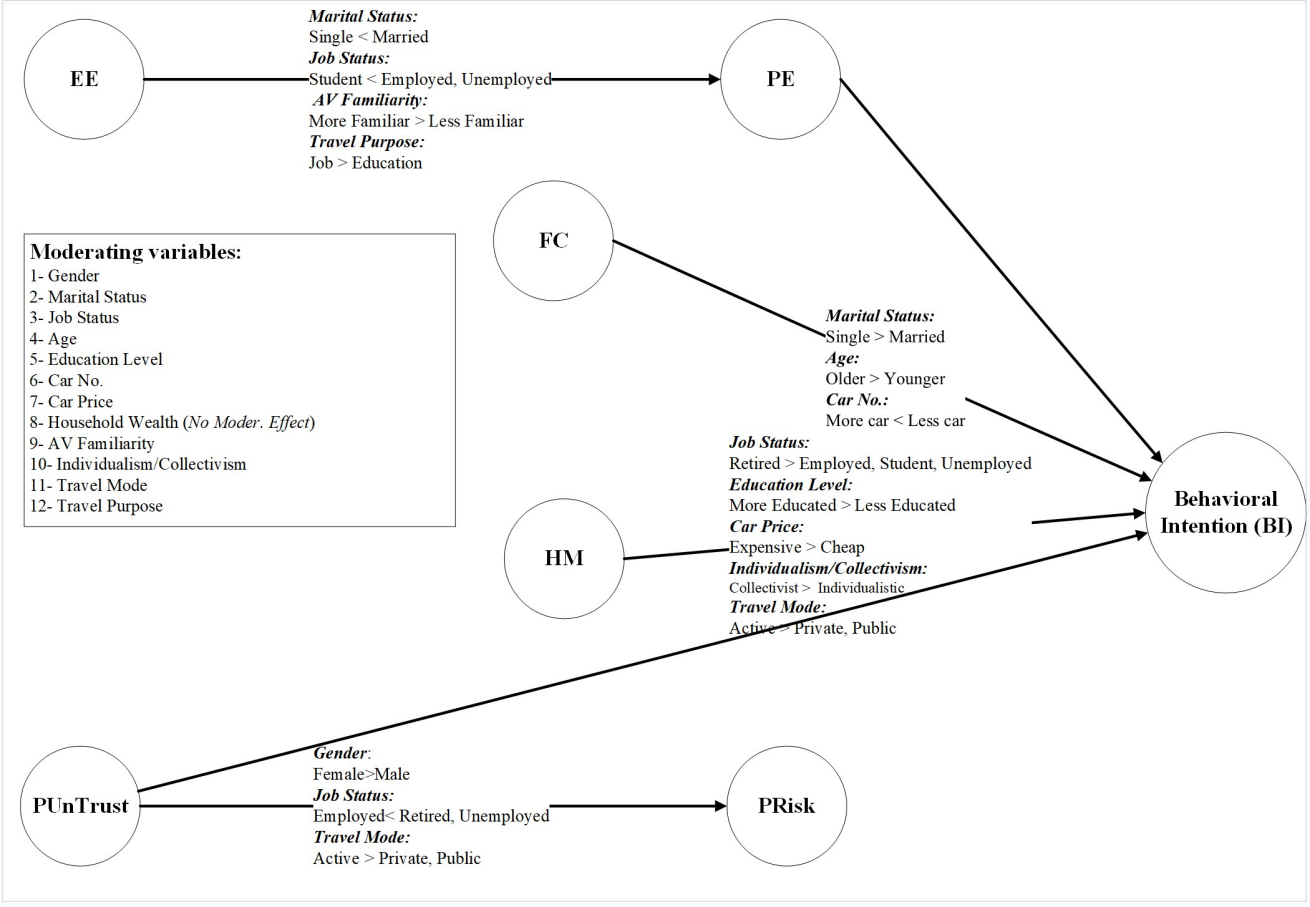
The summary of the effects of moderating variables on the APVAM.

This research has accomplished two significant objectives: developing the APVAM for Tehran citizens and examining a wide range of moderating variables. The findings of the structural equation modeling confirmed six out of the eight hypotheses proposed in this study, while two were rejected. In total, the effects of 12 moderating variables were investigated, which will be discussed in the following sections. Fig 3 summarizes the results of examining the effect of moderator variables on the APVAM.

According to the structural equations modeling results, effort expectancy (EE) positively affected the performance expectancy (PE) of the potential users of APVs (β = 0.481, p-value = 0.000). This result has been in line with the findings of the other studies [8, 9, 29, 33–36]. Among twelve moderating variables examined in this research, marital status, job status, travel mode, travel purpose, and the degree of the previous familiarity with APVs (AV familiarity) had a moderating effect on hypothesis 1.

According to the structural equations modeling results, the effect of the effort expectancy (EE) on behavioral intention (BI) did not prove significant, and hypothesis 2 was rejected (β = 0.036, p-value = 1.072). This result is consistent with the findings of Madigan et al. [25]. This suggests that the extent of ease of use of APVs is not a direct determining factor for the tendency of using such vehicles. Rather, this variable is influential indirectly through the variable of performance expectancy (PE) on behavioral intention (BI).

The results of the structural equation modeling revealed that the most influential factor in explaining behavioral intention (BI) was performance expectancy (PE), confirming hypothesis 3 (β = 0.416, p-value = 0.000). In the study by Venkatesh et al., this variable was also found as the most important construct of the model [16]. Extensive studies have also confirmed the effectiveness of this variable on the tendency to utilizing AVs [8, 9, 22, 25, 29–32, 35]. This suggests that the most important factor for APVs to be welcomed is elaborating the advantages and benefits created through applying this technology for users and clarifying the extent of match between APVs and other modes of transport currently available. The results of investigating the moderating effect of the moderator variables showed that no variable had a moderating effect on the PE → BI path. This suggests that the PE → BI relation has equally high importance for all groups similarly, and the results are not sensitive to changes among different groups.

According to hypothesis 4, the variety and availability of facilities, resources, and conditions of using APVs lead to an increased tendency of people to using this technology. The results of structural equation modeling on APVAM confirmed this hypothesis (β = 0.223, p-value = 0.000). The second variable that predicts the tendency of people to using APVs was facilitating conditions (FC). This concurs with the findings of Madigan et al. who dealt with investigating the acceptance of public AVs [25]. This variable has been the second influential variable on interpreting behavioral intention (BI). The results of examining the moderating effect indicated that three variables of marital status, age, and the number of household cars have a moderating effect on hypothesis 4. For single and older individuals, the degree of FC of applying APVs is significantly more important in their tendency to use such vans compared to married and younger individuals. Also, those who have currently more cars in their household showed less care for this variable in determining their tendency to using APVs.

Hypothesis 5 discusses the extent of the influence of hedonic motivation (HM) on behavioral intention (BI). SEM results showed that according to previous studies, this variable has a significant impact on interpreting the extent of people’s tendency to using APVs [25, 26]. Considering the HM → BI path coefficient (β = 0.184, p-value = 0.000), following PE and FC, this variable has been the third variable explaining the tendency to using APVs. One of the notable points about hypothesis 5 is the high sensitivity of the path coefficient results across different community groups. Among the 12 moderating variables examined in this research, five variables of individualism/collectivism, job status, the widely used vehicle for daily trips, level of education, and the price of the most expensive car of the household had a moderating effect on HM → BI path. According to the moderating effect results, for the individualistic people compared to their collectivist counterparts, the HM variable had a greater impact on explaining BI. Also, the moderating effect results of the variable of job status for this hypothesis indicated that for retired individuals compared to other job states (employed, students, and unemployed), the extent of fun and pleasure of APVs is influential for their tendency to utilizing these vehicles. Indeed, in APVAM, for retired individuals, the HM → BI path coefficient has been 0.5807, which explains the extent of the tendency to APVs more than PE and FC variables. Another effective moderating variable on hypothesis 5 is the widely used means of travel for undertaking the intracity daily trips (travel mode). The results showed that for the individuals who benefit from active transport mode (walking and bicycle) for their daily trips, their extent of perceived fun and pleasure for APVs harms their tendency to using this technology compared to the others (the individuals who commonly used daily travel mode is personal or public transport). Then, upon examining the results for the variables of the level of education and the most expensive car owned by the household, it was found that as the educational level of the subjects increased or the vehicle utilized by their household was more expensive, their perceived fun and pleasure for APVs was more effective on their tendency to utilizing this technology compared to the individuals with lower levels of education and less expensive vehicles owned by the household.

One of the most controversial hypotheses that were investigated in this study was hypothesis 6. According to the SEM results, no relationship was found between perceived risk (PRisk) and behavioral intention (BI), leading to the rejection of hypothesis 6 (β = -0.0008, p-value = 0.0838). This finding aligns with the results of Chen & Yan as well as Choi & Ji [14, 37].

According to hypothesis 7 in APVAM, the degree of the PUnTrust to AVs negatively affected the tendency to utilizing this technology. The results of the SEM confirmed this hypothesis (β = -0.090, p-value = 0.000). Regarding the analysis, as the trust in APVs diminishes, individuals would have less tendency to utilizing this technology [9, 28, 31, 34–36, 38, 39]. Experience has also shown that not welcoming emerging technologies would lead to their failure in marketing. This becomes reinforced or attenuated by the news published about AVs that are in the test stage and investigated by automotive industries. Another notable result obtained from analyzing the moderating effect of the variables was that none of the two variables whose moderating effect on APVAM was studied had a moderating effect on hypothesis 7 (PUnTrust → BI). This suggests the robustness of the obtained results and their equal importance for all segments of society.

The last hypothesis examined in this research was hypothesis 8. According to this hypothesis, the degree of PUnTrust to APVs leads to increased perceived risk towards this technology. This means that the individuals who experienced diminished trust in the efficiency and safety of APVs under the influence of negative news resulting from malfunctioning of these vehicles have greater perceived risk towards these vehicles (β = 0.666, p-value = 0.000) [14, 39, 41]. The results of analyzing the moderating effect indicated that three variables of gender, job status, and commonly used travel mode for the daily trips had a moderating effect on the PUnTrust → PRisk of APVAM. According to the results, the extent of PUnTrust to APVs leads to higher perceived risk among women compared to men. This also applies to retired people compared to employed and unemployed individuals. Also, according to hypothesis 5, there was a significant difference between the path coefficients of PUnTrust → PRisk for the individuals who benefit from active transport mode (cycling and walking) for their daily trips (β = 0.8340) compared to the individuals who use private transport (β = 0.6196) or public transport (β = 0.6798) for their daily travel. Indeed, the level of PUnTrust among individuals who commonly use active transport modes has a greater impact on their perceived risk of AVs. Overall, the results of investigating the moderating effect of travel mode variable on hypotheses 5 and 8 suggest that the individuals who currently use active modes of transport for daily travel do not have an optimistic view of APVs, and they have a negative presupposition to this technology. Table 9 compares the results of investigating the hypotheses of this research against previous studies.

**Table 9:** Comparison of the hypothesis result with previous researches.

Hypothesis	Result	Research with the same results as the present research
**H1: EE→PE**	Confirmed	[8, 9, 29, 33–36]
**H2: EE→BI**	Rejected	[25]
**H3: PE→BI**	Confirmed	[8, 9, 22, 25, 29–32, 35]
**H4: FC→BI**	Confirmed	[25]
**H5: HM→BI**	Confirmed	[25, 26]
**H6: PR→BI**	Rejected	[14, 37]
**H7: PUnTrust →BI**	Confirmed	[9, 28, 31, 34–36, 38, 39]
**H8: PUnTrust →PR**	Confirmed	[14, 39, 41]

## Limitations and future research

This research had the following limitations. First of all, due to the absence of APVs in Iran, participants’ perceptions were solely shaped through news and introductory information about this technology at the beginning of the survey Thus, this may have created a bias in the results. As long as AVs are not provided widely in the market with a reasonable and economical price, expectable volatilities would occur about the public perception [7]. Thus it is suggested that in future studies by providing a sample of these vehicles and creating conditions for a real experience of travel by these vehicles, this bias could be resolved. Secondly, this study employed a cross-sectional approach, which may introduce errors inherent to such study designs. Consequently, it is suggested that future studies adopt a longitudinal approach to minimize potential limitations. Additionally, it is important to note that this study focused solely on citizens of Tehran, who have their own unique cultural and demographic characteristics, while the behavior of other Iranian citizens in other cities may be different based on variables such as the size of the city they live in, the distances of daily trips, plus sociocultural characteristics. Furthermore, the data collection method in this study relied on online surveys, which may inadvertently exclude the attitudes of certain demographic groups affected by the digital divide (e.g., elderly population, individuals with lower digital literacy, or limited access to data networks). Hence, it is recommended that future studies strive to include a larger and more diverse population to account for these potential limitations.

## Conclusions

This study had two primary objectives: 1- developing an acceptance model for APVs, and 2- examining the impact of moderating variables on this model. In order to achieve the first objective, by setting the UTAUT2 model as the basis [25] as well as adding or subtracting some constructs, the APVAM model was developed. The key findings related to the first objective can be prioritized as follows.

- Overall, citizens in Tehran hold a favorable view of using APVs, and their perceived risk associated with these vehicles is relatively low.
- The most effective factor affecting the increase in willingness to use APVs is the performance expectancy (PE). Therefore, focusing on the usefulness of this mode of transportation (such as reducing travel time compared to competing modes) will have the greatest impact on increasing the acceptance of these vehicles. This information should be taken into consideration when developing services that cater to the needs and preferences of potential users of APVs in the Tehran Metropolitan area.
- Understanding the acceptance levels of APVs among the public is crucial. The research uncovered the factors that influence the willingness of citizens in Tehran to accept this mode of transportation. It was discovered that performance expectancy (PE), facilitating conditions (FC), and hedonic motivation (HM) have a positive impact, while perceived Un-Trust (PUnTrust) has a negative effect on the inclination to use APVs among Tehran citizens.
- It was also found that effort expectancy (EE) does not directly influence the tendency of individuals to utilize APVs. However, it indirectly affects their tendency to utilize these vehicles through performance expectancy (PE) by increasing their likelihood of doing so.
- The most effective factor affecting the increase in willingness to use APVs is the performance expectancy (PE). Therefore, focusing on the usefulness of this mode of transportation (such as reducing travel time compared to competing modes) will have the greatest impact on increasing the acceptance of these vehicles. This information should be taken into consideration when developing services that cater to the needs and preferences of potential users of APVs in the Tehran Metropolitan area.

Then, the effect of 12 moderating variables was investigated on APVAM. These 12 variables included individualism/collectivism, gender, marital status, job status, the commonly used vehicle for intracity travel, the major purpose of intracity travel, age, level of education, degree of the previous familiarity with AVs, the number of household cars, the price of the most expensive household car, and household wealth. The results of all these analyses were discussed thoroughly in previous sections. Nevertheless, the most important results obtained from examining the effect of moderating variables on the APVAM can be summarized as follows.

- No variable had a moderating effect on the PE → BI and the PUnTrust →BI paths and the results have low sensitivity to changes across different groups.
- For single and older individuals, the degree of FC of applying APVs is significantly more influential to use APVs compared to married and younger individuals. For individuals who rely on personal or public transportation for the majority of their daily trips, as opposed to those who primarily use active transport modes like walking or cycling, the perceived enjoyment of APVs plays a more significant role in increasing their willingness to use these vehicles. These findings should be taken into account in potential demand estimation studies for APVs in the Tehran Metropolitan area. For the individualistic people compared to their collectivist counterparts, the HM variable had a greater impact on intention to use APVs.

## Supporting information

S1 File Raw data. The minimal data set.
(XLSX)

S2 File SEM modeling. SmartPLS software version 3 or higher is required to view the codes.
(ZIP)
